# Breast and ovarian cancers: toward a multi-cancer early detection test

**DOI:** 10.3389/fimmu.2026.1741582

**Published:** 2026-03-19

**Authors:** Azza Habel, Maryem Bessaad, Mouna stayoussef, Weili Xu, Hanen Bouaziz, Mouna Ayadi, Anis Larbi, Basma Yaacoubi-Loueslati

**Affiliations:** 1 Laboratory of Mycology, Pathologies, and Biomarkers (LR16ES05), Faculty of Sciences of Tunis, University of Tunis El Manar, Tunis, Tunisia; 2 Singapore Immunology Network (SIgN), Agency for Science Technology and Research (A*STAR), Singapore, Singapore; 3 Salah Azaiez Oncology Institute, Tunis, Tunisia; 4 Beckman Coulter Life Sciences, Villepinte, France

**Keywords:** breast cancer, liquids biomarkers, liquids biopsie, multi-cancer early detection test, ovarian cancers

## Abstract

**Introduction:**

Breast cancer (BC) and ovarian cancer (OC) are leading causes of cancer-related mortality among women worldwide. Despite their distinct clinical presentation these malignancies share genetic, hormonal, and micro-environmental characteristics, suggesting overlapping mechanisms of tumorigenesis and immune escape. Identifying common immune-related biomarkers could improve large scale early detection and guide the development of shared therapeutic strategies.

**Methods:**

We conducted a multiplex immunoassay profiling of 81 immune-related proteins including cytokines, chemokines, growth factors, and immune checkpoints in serum samples from 57 BC patients, 57 OC patients, and 49 healthy controls (HC).

**Results:**

Multivariate and univariate analyses were utilized to identify proteins exhibiting significant dysregulation. Receiver Operating Characteristic (ROC) curve analyses were conducted to evaluate the diagnostic performance of individual and combined protein panels. Protein-protein interaction networks were constructed using STRING and Cytoscape to elucidate shared functional modules. Multivariate analysis revealed a partial yet significant distinction between cancer patients and HC, with BC and OC exhibiting notable immunological similarities. Eighteen proteins were dysregulated in BC and OC as compared to HC, indicating shared oncogenic and immunological pathways. Furthermore, individual biomarkers exhibited moderate diagnostic performance, while combined panels achieved high accuracy. Network analysis revealed highly interconnected modules centered on the TNF/TNFR superfamily, co-stimulatory molecules, and chemokines, suggesting promising targets for combinatorial immunotherapy.

**Disscussion:**

our findings demonstrate a significant overlap in immune-related protein expression between BC and OC, supporting the feasibility of a common diagnostic biomarker panel for these female cancers. The identified proteins and their interaction networks offer valuable insights into the shared mechanisms of tumor progression and immune evasion, presenting promising avenues for early diagnosis and targeted therapy.

## Introductions

Globally, breast cancer (BC) is the most prevalent type of malignancy and the leading cause of cancer-related mortality among women. In contrast, while ovarian cancer (OC) is relatively rare, it ranks as the leading cause of gynecological cancer-related deaths worldwide. In fact, OC is one-tenth as common as BC but is three times more lethal (1).

Ovarian and breast cancers share several characteristics, including specific genetic mutations (such as tumor suppressors and proto-oncogenes), alterations changes in hormone regulation and a complex microenvironment (2). Numerous studies have sought to uncover the relationships between BC and OC (2–5). Understanding the correlations between different types of cancers enhances our knowledge of the disease process and opens the possibility of developing a unique non-invasive test based on the detection of a shared panel of biomarkers associated with an increased risk for both BC and OC types.

Recent advances in molecular oncology have uncovered overlapping pathways involved in tumor development, including chronic inflammation within the tumor microenvironment (TME) and dysregulated immune responses, which contribute to this process (6).

Tumor development, immune evasion, and resistance to treatment are significantly influenced by the TME, which comprises immune cells, stromal cells, extracellular matrix components, and signaling molecules. Key mediators within the TME include cytokines, chemokines, growth factors, and immune checkpoint molecules, which orchestrate immune surveillance, promote tumor angiogenesis, and contribute to the dynamic crosstalk between cancer cells and their environment (6, 7).

In light of the crucial role played by the tumor microenvironment and immune-related pathways in cancer progression, there is a growing interest in identifying circulating biomarkers that reflect this complex biological interplay. Traditional diagnostic approaches based on single or limited biomarkers are increasingly being supplanted by multiplex strategies capable of capturing the molecular heterogeneity of tumors. Multi-Cancer Early Detection (MCED) tests exemplify this paradigm shift by enabling the detection of multiple cancers through non-invasive blood-based profiling. However, most existing MCED platforms rely on a limited number of analytes, often focusing on genomic or epigenetic markers, with relatively few incorporating immune-related proteins.

In this context, we propose a comprehensive proteomic approach that quantifies 81 immune-related mediators in the serum of HC, BC, and OC patients. This approach aims to better characterize the molecular landscape shared by breast and ovarian cancers and to support the future development of robust, protein-driven early detection tools tailored to these tumor types.

More specifically, this study seeks to contribute to the emerging concept of *Multi-Cancer Early Detection (MCED)* by evaluating a common circulating protein signature capable of detecting both BC and OC at early stages. Additionally, we aim to highlight common signaling pathways involved in their progression, which serve as potential shared therapeutic targets.

## Patients and methods

### Patients

Fifty-seven female patients with OC, and BC were recruited from the outpatient surgical and oncology service of the Salah Azaiez Institute (SAI) between March 2019 and November 2020. All OC and BC patients are newly diagnosed and confirmed through histopathological examination. At the time of recruitment, none of the patients had received any therapy. The clinicopathological characteristics of the patients were obtained from medical records and structured interviews using a questionnaire.

Forty-nine age-matched healthy women without any history of cancer were included as HC. None of the participants had any unrelated diseases. All patients and controls provided written informed consent. All documents and procedures were monitored and approved by the Research and Ethics Committee of SAI (registration number: SAI/2019/01, granted February 22, 2019). The study was conducted in accordance with the ethical standards outlined in the 1964 Declaration of Helsinki.

### Blood collection

Five milliliters of venous blood are collected in a sterile serum coagulation tube. Serum samples are separated within two hours of collection by centrifugation at 2.500 rpm for 20 minutes. They are then transferred to a clean microcentrifuge tube and centrifuged at 14.000 rpm for 10 minutes to remove cellular debris and fragments. The serum samples are aliquoted and stored at -80°C until analysis.

### Multiplex immunoassay

We measured the concentrations of 81 target proteins in serum, which included 33 cytokines (G-CSF, GM-CSF, IFN-α, IFN-γ, IL-1α, IL-1β, IL-2, IL-3, IL-4, IL-5, IL-6, IL-7, IL-8, IL-9, IL-10, IL-12p70, IL-13, IL-15, IL-16, IL-17A, IL-18, IL-20, IL-21, IL-22, IL-23, IL-27, IL-31, LIF, M-CSF, MIF, TNF-α, TNF-β, and TSLP), 18 chemokines (BLC, ENA-78, eotaxin-1, eotaxin-2, eotaxin-3, fractalkines, GROα, IP-10, I-TAC, MCP-1, MCP-2, MCP-3, MDC, MIG, MIP-1α, MIP-1β, MIP-3α, and SDF-1α), 6 growth factors/regulators (FGF-2, HGF, MMP-1, NGF-β, SCF, VEGF-A), and 8 soluble receptors (APRIL, BAFF, CD30, CD40L, IL-2R, TNF-RII, TRAIL, and TWEAK) using the ProcartaPlex Human Immune Monitoring 65-Plex Panel (Invitrogen) and 16 Immunes checkpoints proteins: (BTLA, CD27, CD28, CD40, CD80/B7-1, CD86/B7-2, CTLA-4, GITR, GITRL, HVEM, ICOS, LAG-3, PD-1, PD-L1, TIM-3, and TLR2), using the MILLIPLEX MAP^®^ Human Immuno-Oncology Checkpoint Protein Magnetic Bead Panel (Millipore, Billerica, MA, USA) according to the manufacturer’s protocol. Data were collected using a Bio-Plex^®^ 200 instrument and analyzed with Manager 5.0 software (Bio-Rad, Hercules, CA, USA).

### Statistical analysis

All statistical analyses were conducted using R software version 4.4.3.

Data preprocessing and normalization: Protein concentration values were log2-transformed to reduce heteroscedasticity and improve normality. Data normalization was applied to minimize technical variability across samples and ensure comparability between groups.

Univariate analyses: The normality of each protein’s distribution was assessed using the Shapiro-Wilk test. Non-parametric comparisons between groups were performed using the Wilcoxon rank-sum test. To account for multiple testing across the 81 proteins, false discovery rate (FDR) adjustment using the Benjamini-Hochberg method was applied in all univariate analyses to control for type I errors. Proteins with adjusted p-values (FDR) < 0.05 were considered statistically significant.

Multivariate analyses: Principal Component Analysis (PCA) and sparse Partial Least Squares Discriminant Analysis (sPLS-DA) were employed to assess global expression patterns and to evaluate the capacity of the protein profiles to differentiate between BC, OC, and HC groups. Diagnostic performance: ROC curve analysis was then conducted to evaluate the diagnostic relevance of individual proteins and to assess the combined discriminatory power of multiple proteins. The sensitivity and specificity of each biomarker were evaluated using the area under the curve (AUC) as a measure.

Pathway analysis: the most relevant proteins identified through ROC analysis were utilized for pathway enrichment analysis to explore common signaling pathways potentially involved in the development of both BC and OC. This step was conducted using R (v4.4.3), as well as STRING and Cytoscape software, to visualize and interpret shared molecular networks and functional annotations.

## Results

### Study subjects

We compared the serum protein expression profiles of 81 proteins among HC (N = 49), BC (N = 57), and OC patients (N = 57) to identify immune mediator signatures in serum associated with the risk of BC and OC. Additionally, we explored potential of these signatures as early diagnostic biomarkers for these cancers.

**Table 1** summarizes the clinical and pathological data. The average age of BC patients was 52 years, while OC patients had an average age of 53 years. The mean body mass index (BMI) was 29 ± 5.15 for BC and 26.4 ± 6.8 for OC patients. According to the International Federation of Gynecology and Obstetrics (FIGO), 29 (50.88%) had late-stage BC (stage III and stage IV), compared to 28 (49.12%) BC patients diagnosed at an early stage. Additionally, nine (15.79%) patients had early-stage OC (stage I and stage II) while 48 (84.21%) had late-stage OC (stage III and stage IV). Furthermore, 31 BC patients (54.39) and 30 OC patients (52.64%) had distant metastases (**Table 1**).

**Table 1:** Clinical data of healthy controls, BC and OC patients.

	Characteristic		N(%)
Healthy controls Characteristics (N = 49)	Means Age (yr) (± SD)		56 ± 13
	BMI (kg/m²) (± SD)		27.66 ± 4.5
BC Characteristics (N = 57)	Means Age (yr) (± SD)		53 ± 11
	BMI (kg/m²) (± SD)		26 (45.61)
	Distant Metastasis	M0	29 ± 5.15
		M1	31 (54.39)
	FIGO Staging	Early-stage	28 (49.12)
		Late-stage	29 (50.88)
	Grade	Low grade	34(59.65)
		High grade	23 (40.35)
	TNBC	NTNBC	49 (85.96)
		TNBC	8 (14.04)
	RE	RE-	13 (22.81)
		RE+	44 (77.19)
	RP	RP-	18 (31.58)
		RP+	39 (68.42)
OC Characteristics (N = 57)	Means Age (yr) (± SD)		52 ± 18
	BMI (kg/m²) (± SD)		26.4 ± 6.8
	Distant Metastasis	M0	27 (47.36)
		M1	30 (52.64)
	FIGO Staging	Early-stage	9 (15.79)
		Late-stage	48 (84.21)
	Grade	Low grade	36(63.15)
		High grade	19 (36.85)

N, number of the population. Yr, years; SD, standard deviation; BMI, Body Mass Index; BC, Breast Cancer; OC, Ovarian Cancer; FIGO, International Federation of Gynaecology and Obstetrics; TNBC, Triple negative-BC; NTNBC -NO Triple negative BC RE, Receptor of Oestrogen; RP, Receptor of Progesterone.

### Discrimination of groups by multivariate analysis

A multivariate analysis approach was employed to examine the overall profiles of the 81 immune mediators measured in HC, BC, and OC patients.

**Figure 1:**
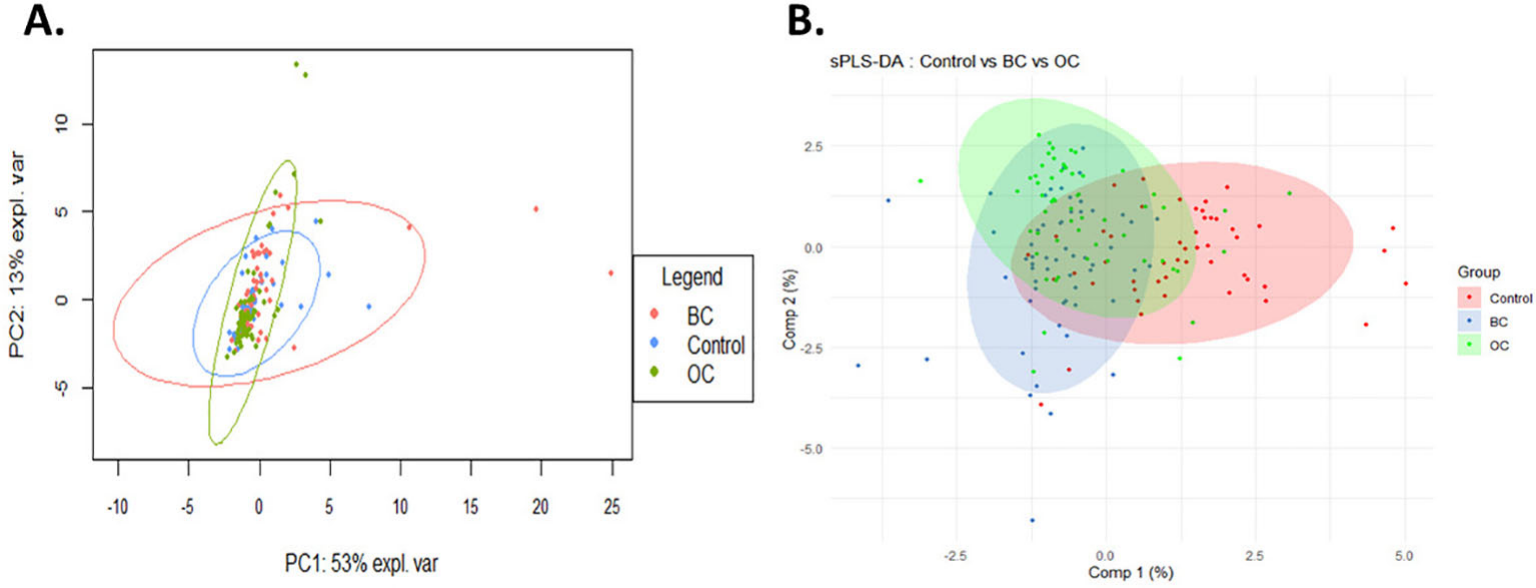
Multivariate analysis of prognostic profiles in patients with BC, OC, and healthy controls. Principal component analysis revealed a partial yet clear separation between HC, BC, and OC groups, with PC1 and PC2 explaining 53% and 13% of the total variance, respectively **(A)**. Subsequent sparse partial least squares discriminant analysis improved the discrimination between groups, highlighting distinct immune profiles in cancer patients compared to HC, and revealing immunological similarities between BC and OC **(B)**.

Principal component analysis revealed a partial yet distinct separation among the three groups, with the first principal component (PC1) accounting for 53% of the total variance and the second principal component (PC2) 13% (**Figure 1A**). This distribution suggests a structuring of the data based on disease status. To enhance the discrimination between groups, a sPLS-DA was performed (**Figure 1B**). This approach facilitates the separation of HC, BC, and OC groups by identifying the directions that maximize intergroup differences. The clear grouping of observations according to their respective categories supports hypothesis of distinct immune profiles between cancer patients and HC, and reflect a partial overlap between BC and OC.

### Hierarchical clustering and heatmap visualization

**Figure 2:**
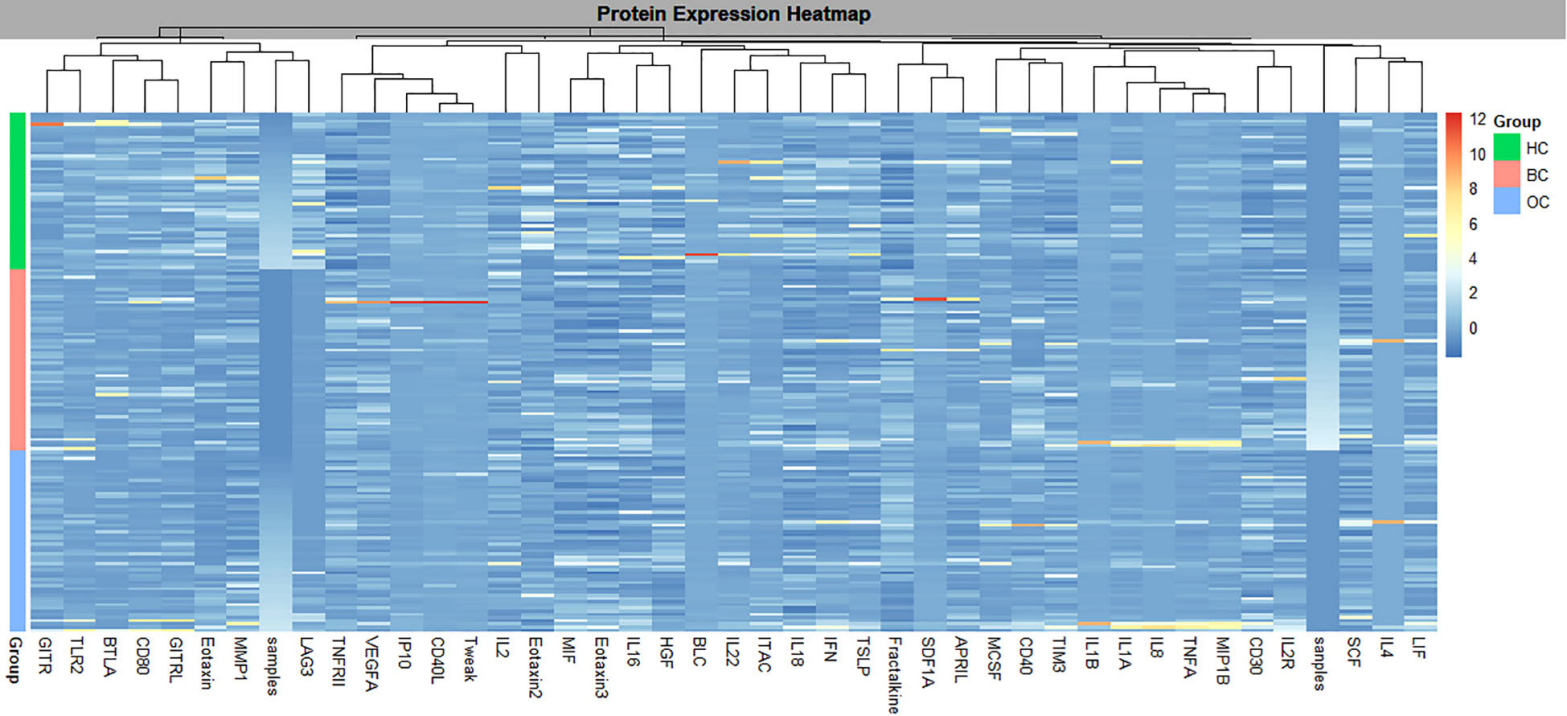
Heatmap of differentially expressed proteins between Control, BC, and/or OC groups. Global protein expression profiling of BC, OC, and HC groups showed distinct proteomic patterns between cancer patients and healthy controls. Several proteins displayed similar dysregulation in BC and OC compared to HC. Elevated levels of VEGF-A, TWEAK, IL-6, and TNF-α were detected, indicating activation of inflammatory, immunoregulatory, and pro-angiogenic pathways.

The global protein expression analysis of the BC, OC, and HC groups revealed distinct proteomic profiles between cancer patients and HC. Notably, several proteins exhibited similar dysregulation patterns in both types of cancers compared to HC, indicating shared molecular alterations. The results demonstrated increased levels of certain proteins including VEGF-A, Tweak, IL-6, and TNF-α, suggesting the activation of inflammatory, immunoregulatory, and pro-angiogenic pathways. These similarities support the potential development of a shared biomarker panel for cross-diagnostic or monitoring purposes in gynecologic cancers (**Figure 2**).

**Figure 3:**
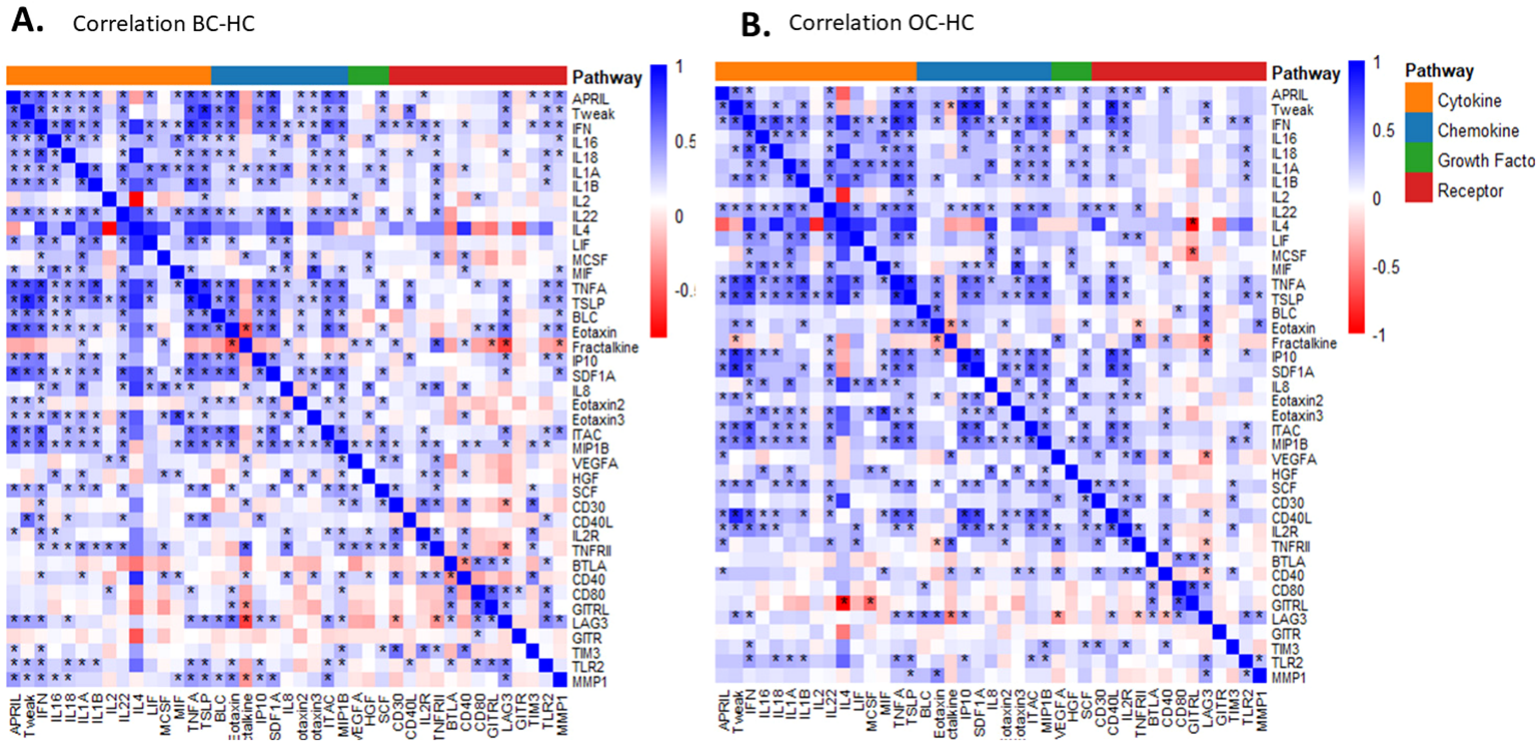
Heatmaps of differentially expressed proteins grouped by functional category in BC and OC. Correlation heatmaps illustrating distinct protein co-regulation modules in BC vs HC **(A)** and OC vs HC **(B)**. In BC vs HC, a prominent inflammatory cluster is observed, including cytokines (IL-1β, IL-6, IL-18, IL-22), growth factors (VEGFA, SCF), and chemokines (BLC, Eotaxin, Fractalkine, SDF1A) showing strong positive correlations. In OC vs HC, similar cytokine–growth factor associations are present, together with specific modules involving immune receptors (CD40L, BTLA, CD80, GITR, LAG3, TIM3).

The comparative analysis of the correlation heatmaps (**Figure 3**) reveals distinct protein co-regulation modules and notable similarities among the BC vs HC, OC vs HC, and BC vs OC groups. In the BC vs HC comparison (**Figure 3A**), a prominently marked inflammatory cluster is observed, which includes cytokines such as IL-1β, IL-6, IL-18, IL-22, as well as growth factors like VEGFA and SCF. These proteins exhibit strong positive correlations, indicating a coordinated orchestration of the inflammatory and angiogenic responses in BC. Additionally, chemokines such as BLC, Eotaxin, Fractalkine, and SDF1A are integrated into this network, reinforcing the concept of an active inflammatory tumor microenvironment.

In the comparison of OC vs HC (**Figure 3B**), there are similarities with BC vs HC, particularly the positive correlations between inflammatory cytokines (IL-1β, IL-6, IL-18) and growth factors (VEGFA, SCF). This suggests that the activation of these pathways is a common characteristic of both types of cancer when compared to healthy controls. However, the heatmap for OC vs HC reveals specific modules involving immune receptors such as CD40L, BTLA, CD80, GITR, LAG3, and TIM3, indicating a more pronounced engagement of immunosuppressive pathways in OC. This profile reflects a tumor microenvironment where immune evasion mechanisms are particularly active.

The comparison between BC and OC provides a more nuanced distinction of the specificities associated with each tumor type. While some inflammatory modules persist, the co-regulation of immune checkpoint proteins particularly LAG3, TIM3 and GITR is significantly stronger in the OC group. In contrast, the inflammatory cluster which centers around IL-1β, IL-6 and MIF is more characteristic of the BC group. This heatmap thus underscores the presence of two major signaling axes: that is inflammatory in BC, and the other immunosuppressive and dominant in OC.

### Univariate analysis of immune mediators

A comparative analysis of protein profiles among the HC, BC and OC groups identified a total of 42 proteins that were significantly expressed in patients with BC and/or OC compared to HC (See **Supplementary Data 1**).

**Figure 4:**
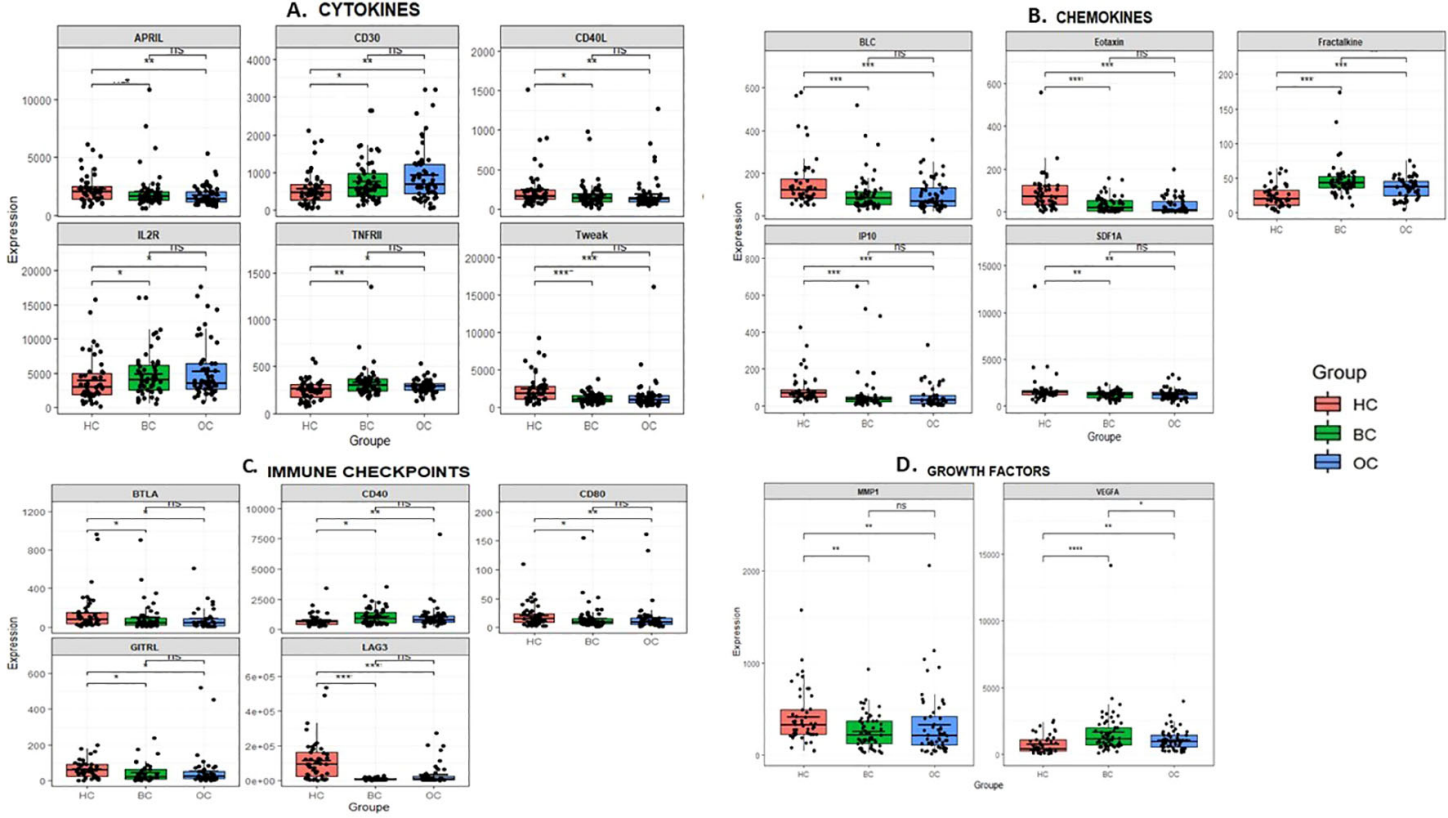
Proteins differentially expressed in BC and OC. Among the quantified proteins, 18 showed significant differences in both BC vs HC and OC vs HC comparisons These include 6 cytokines (APRIL, CD30, CD40L, IL-2R, TNFRII, TWEAK), 5 chemokines (BLC, Eotaxin, Fractalkine, IP-10, SDF-1α), 5 receptors (BTLA, CD40, CD80/B7-1, GITRL, LAG-3), and 2 growth factors (VEGF-A, MMP-1). Most were upregulated in both cancer groups relative to HC, such as Fractalkine (BC: 47.23 pg/ml, OC: 36.16 pg/ml, HC: 24.22 pg/ml) and VEGF-A (BC: 1633.47 pg/ml, OC: 1054.32 pg/ml, HC: 717.06 pg/ml). In contrast, some proteins, including MMP-1 (BC: 251.03 pg/ml, OC: 322.28 pg/ml, HC: 412.09 pg/ml) and BTLA (BC: 142.74 pg/ml, OC: 100.53 pg/ml, HC: 84.33 pg/ml), showed lower levels in BC compared to HC. BC, Breast cancer; OC, Ovarian cancer; HC, Healthy controls, *pvalue >0.05, **pvalue >0.001, ***pvalue >0.0001.

Among these proteins, 18 exhibit significant differences between BC and HC as well as between OC and HC. This group includes 6 cytokines (APRIL, CD30, CD40L, IL-2R, TNFRII, Tweak), 5 chimokines (BLC, Eotaxin, Fractalkine, IP-10, SDF-1alpha), 5 receptors (BTLA, CD40, CD80/B7-1, GITRL, LAG-3), and 2 growth factors (VEGF-A, MMP-1) indicating a common regulatory mechanism in these two tumor types (**Figure 4**).

The majority of these proteins were upregulated in both cancer groups compared to HC. For example, Fractalkine levels increased in BC (47.23 pg/ml) and OC (36.16 pg/ml) compared to HC (24.22 pg/ml), and VEGF-A in BC (1633.47 pg/ml) and OC (1054.32 pg/ml) compared to HC (717.06 pg/ml). However, some proteins, such as MMP-1 (BC: 251.031 pg/ml, OC: 322.275 pg/ml vs 412.09pg/ml in HC) and BTLA (BC: 142.74 pg/ml, OC: 100.53 pg/ml vs 84.33 pg/ml in HC), were slightly less expressed in BC than in HC, suggesting complex regulatory mechanisms.

A group of 28 proteins exhibited significantly different expression levels between BC and OC, although several were altered when compared to HC. These proteins included various pro-inflammatory cytokines (IFN-gamma, IL-1alpha, IL-1beta, IL-12p70), chemokines (IL-8, Eotaxin-3, MIP-1beta), growth factors (HGF, M-CSF), as well as receptors and immune checkpoints (TIM-3, LAG-3, TLR-2). Notably, there was a general trend of overexpression in BC as compared to OC for certain inflammatory cytokines, such as IL-8 and IFN-gamma. While Immune checkpoints such as LAG-3 and TIM-3 are particularly elevated, especially in OC, which may indicate a more pronounced local immunosuppression in this type of cancer (see **Supplementary Data 1**).

### ROC curve analysis and assessment of biomarker discriminatory power

**Figure 5:**
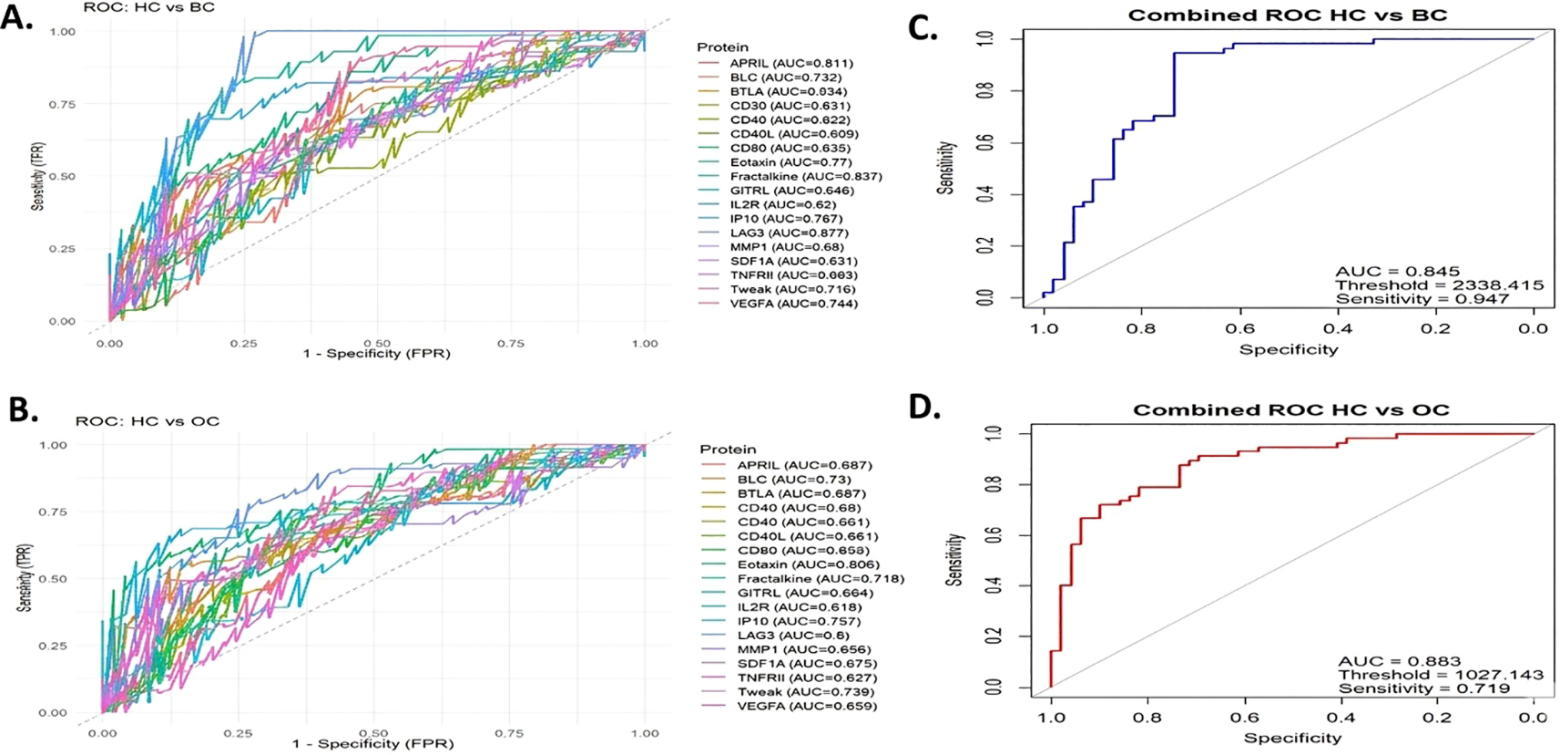
ROC curve analyses of differentially expressed proteins. ROC curve analyses of significantly different immune-related proteins. In HC vs BC **(A)**, highest AUC values were observed for LAG3 (0.877), Fractalkine (0.837), and Eotaxin (0.77), while APRIL (0.611) and CD40L (0.609) showed lower performance. In HC vs OC **(B)**, Eotaxin (0.806), LAG3 (0.800), and IP-10 (0.757) were the top markers. Multivariate ROC models combining all significant proteins improved discrimination, with HC vs BC **(C)** reaching an AUC of 0.845 and sensitivity of 94%, and HC vs OC **(D)** reaching an AUC of 0.883 and sensitivity of 71.9%. BC, Breast cancer; OC, Ovarian cancer; HC, Healthy controls; ROC, Receiver Operating Characteristic; AUC, area under the curve.

To evaluate the discriminatory potential of immune-related proteins that were found to be significantly different through the Mann–Whitney test, individual ROC curve analyses were performed for each comparison group. In distinguishing between HC and BC, the top-performing biomarkers included LAG3 (AUC = 0.877), Fractalkine (AUC = 0.837), and Eotaxin (AUC = 0.77), demonstrating excellent discriminatory capacity. However, some proteins such as APRIL (AUC = 0.611) and CD40L (AUC = 0.609) exhibited more modest performance (**Figure 5A**). Regarding discrimination between HC and OC patients, Eotaxin emerged as the best-performing biomarker (AUC = 0.806), followed closely by LAG3 (AUC = 0.8) and IP10 (AUC = 0.757) (**Figure 5B**).

Subsequently, a multivariate ROC analysis was performed by integrating all significantly different proteins into a single model. This approach enhanced the overall discriminative power. In the comparison between HC and BC, the combined model achieved an AUC of 0.845 with a sensitivity of 0.94, suggesting promising potential for distinguishing BC based on immune profiling (**Figure 5C**). Similarly, in the comparison between HC and OC, the multivariate model yielded an AUC of 0.883 and a sensitivity of 0.719, indicating encouraging but slightly lower discriminative ability compared to the BC group (**Figure 5D**).

### Protein–protein interaction network analysis using STRING

**Figure 6:**
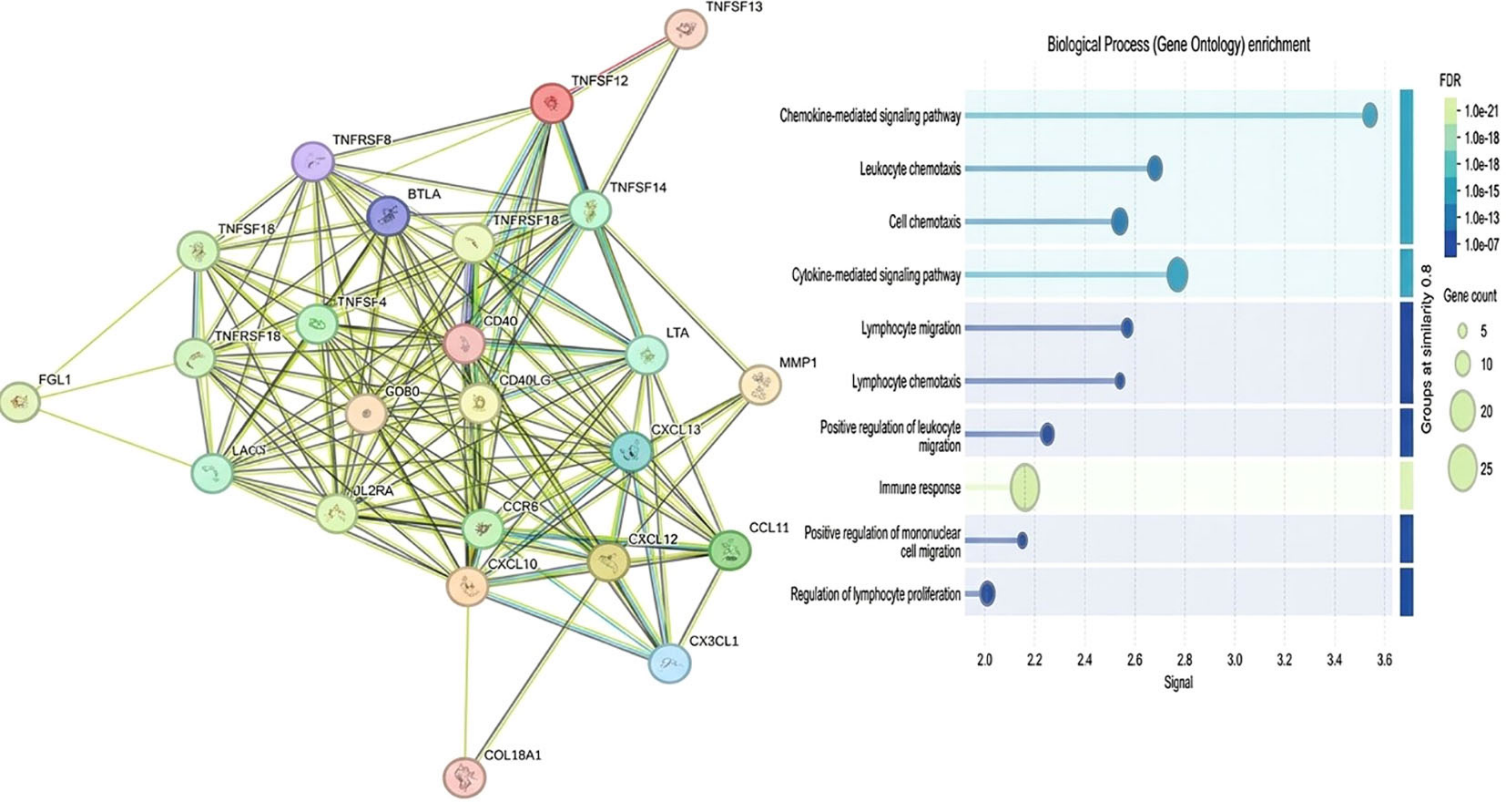
Network mapping of differentially expressed proteins and enriched biological processes in BC and OC. Protein–protein interaction network of proteins commonly identified in BC and OC groups. The STRING-based network shows several distinct functional clusters, including the TNF/TNFR superfamily (TWEAK, TNFα, GITRL), co-stimulatory molecules (CD40, CD80, CD40LG), and chemokines (SDF-1A, Eotaxin-1, BLC). TWEAK serves as a central hub with multiple high-confidence interactions. Other proteins, including BTLA, LAG3, and IL2RA, are organized into a sub-cluster associated with immune evasion, highlighting their coordinated roles in inflammatory and immune regulatory pathways.

The protein–protein interaction network generated from commonly identified proteins between the BC and OC groups reveals a highly interconnected functional structure. The graph, constructed using the STRING database, reveal several distinct functional clusters, including the TNF/TNFR superfamily (TWEAK, TNFα, GITRL), co-stimulatory molecules (CD40, CD80, CD40LG), and chemokines (SDF-1A, Eotaxin-1, BLC). TWEAK displayed multiple predicted high-confidence interactions within the network, indicating a potentially prominent position in the inferred interaction landscape. Other proteins, such as BTLA, LAG3, and IL2RA are integrated within a sub-cluster associated with immune evasion, suggesting their involvement in modulating anti-tumor immune responses. This network may help identify proteins of interest that could serve as potential candidates for future investigation in combinatorial immuno-oncology strategies (**Figure 6**).

## Discussion

Cancer, with its far-reaching impact on individuals and societies, represents a significant global health challenge that affects millions of people worldwide (1). Numerous studies have shown the remarkable similarities that exist between OC and BC, including altered hormone regulation, a complex microenvironment, and comparable genetic alterations involving tumor suppressors and proto-oncogenes (2, 3). Additionally, it has been reported that BC (8, 9) and OC (10–12) can be associated with immune dysfunction, which fails to recognize malignant cells and prevents their successful elimination during apoptosis, thereby implicating various immune mediators.

The similarities between the two types of malignancies and their ability to utilize immune mediators such as cytokines, chemokines, and immune checkpoints as mechanisms of immune escape have prompted researchers to explore common tumorigenic pathways. The goal is to develop a multi-cancer early detection test based on a shared panel of immune-related biomarkers (13–15).

In the present study, we explored the immunological landscape of BC and OC by simultaneously profiling 81 immune-related proteins. We employed both multivariate and univariate approaches, in the aim to identify distinct and/or shared expression patterns between the two cancer types.

Several recent studies have investigated multiplex biomarker techniques in cancer diagnosis. For example, a Chinese study utilized a panel of immune mediators to differentiate between triple-negative breast cancer and benign lesions, achieving a sensitivity of 82% and a specificity of 75%. Similarly, an 11-biomarker panel was validated for detecting OC with high diagnostic accuracy. Compared to previous efforts, our analysis encompasses a broader spectrum of proteins, incorporating both tumor-derived and immune-related markers, which may enhance the panel’s robustness and generalizability across various cancer subtypes.

In our study, we identified a panel of 18 proteins that are dysregulated in both BC and OC when compared to HC demonstrates the presence of overlapping oncogenic and immunological pathways between these two types of cancer. Notably, proteins such as CX3CL1, VEGF-A, BLC, Eotaxin, CXCL10, and SDF-1, along with immune checkpoint markers including CD30, CD40, CD80/B7-1, LAG-3, BTLA, GITRL, APRIL, and CD40L, were found to be expressed similarly in both cancer types. These immune mediators could be potentially involved in processes such as immune regulation, angiogenesis, and tumor microenvironment remodeling hallmarks of cancer biology (16, 17).

The convergence of these protein signatures suggests that BC and OC may share common mechanisms of tumor progression, particularly those related to immune evasion and chronic inflammation. Recent studies utilizing artificial intelligence technologies have highlighted shared molecular mechanisms and biomarkers across BC and OC (14, 18). For example, chemokines such as IP-10 and SDF-1alpha are known to mediate immune cell trafficking and tumor-stroma interactions (19), while VEGF-A serves as central driver of angiogenesis in both BC and OC (20, 21). The convergence of these proteins in both cancers supports the hypothesis of shared oncogenic and immunomodulatory pathways, as suggested by network and clustering analyses in large-scale studies (14, 18).

Furthermore, the use of broad proteomic panels has demonstrated that multivariate models can achieve high diagnostic accuracy, exceeding surpass in traditional single markers such as CA-125 (22, 23).

Our ROC analysis supports this finding, revealing that while individual proteins exhibited significant discriminative power (AUC 0.6-0.8), the combined panel achieved an AUC greater than 0.88, suggesting the potential added value of multiplexing for distinguishing cancer cases.

### Biological significance of shared and distinct biomarkers

Ovarian cancer exhibited a significant downregulation of various pro-inflammatory and regulatory cytokines, including VEGF, IFN-γ, IL-12p70, IL-1β, IL-2, IL-21 and TNF-α. This pattern aligns with the more immunosuppressed and less inflammatory microenvironment characteristic of OC (24). Furthermore, the upregulation of TIM-3 in OC compared to BC might contribute to T cell exhaustion and immune evasion in ovarian tumors (24). Such differences may be attributed to anatomical factors such as, the peritoneal environment in OC, distinct genetic drivers, and tumor heterogeneity (22, 25).

To investigate the biological significance of the common biomarkers between BC and OC, we used STRING to perform a protein-protein interaction (PPI) analysis. The resulting network suggests the presence of a highly interconnected module enriched with immunological and matrix-related proteins, underscoring their coordinated involvement in tumor progression mechanisms.

This network’s include members of the TNF superfamily (April, GITR, CD40, CD30), costimulatory molecules (CD40LG, CD80), matrix remodeling proteins (COL1A1, COL1A2, MMP1), and immunological regulators such as SDF1A and CCR5. These proteins could play key roles in inflammation, T-cell activation, immune evasion, and extracellular matrix (ECM) remodeling, all of which have been associated to tumor growth and metastatic potential (26).

Interestingly, this network structure aligns with other PPI-based analyses in cancer. A previous study conducted on high-grade serous OC found a similar abundance of TNF-superfamily members and collagen-related proteins, highlighting the potential involvement of these proteins in immune suppression and the development of colorectal, head and neck cancers (27). Similarly, in the comparative network analysis of BC, Fraktaline and SDF1A were identified as proteins shared among different types of metastases, suggesting the possible roles in cell migration and invasion (28). Furthermore, the overexpression of the CD40/CD40L axis in several cancers including BC and OC has been associated with a poor prognosis due to its dual role in immune cell recruitment and tumor cell proliferation and CD40L was proposed as a biomarker for cancer diagnosis (29).

The observed interactions between immune checkpoint-related receptors (TNFRSF18, TNFRSF4) and chemokine pathways (CXCL12, CCR5) further support an immune escape phenotype, which has been well documented in both OC and BC. In fact, CXCL12–CXCR4/CCR5 signaling has been implicated in tumor homing and organ-specific metastasis in both types of cancer (30, 31), offering a potential mechanistic explanation for our findings.

Overall, the significant interconnectedness of our network and the presence of these proteins highlight their potential role as a candidates for further functional investigations in the tumor microenvironment.

These findings support the selection of biomarker candidates based on independent interaction evidence in a hypothesis-generating context and suggest the presence of shared molecular features OC and BC. Such similarity indicates common vulnerabilities that could be exploited for biomarker-based screening or targeted therapy.

Future research should prioritize validating the most significant relationships, particularly the CD40-CD40L-TNFSF13 axis and the CXCL12-COL1A1-CCR5 group, through co-expression analysis and laboratory-based functional investigations in cancer models. Integrating these findings with transcriptomic or epigenomic data may enhance the accuracy of biomarker panels and facilitate the discovery of novel combination therapeutic targets.

### Limitations

This study is retrospective and includes a moderate sample size, which may limit generalizability. An important limitation of this study is the lack of information on tumor stage and metastatic status, as well as the imbalance in disease stage between BC and OC cohorts. Consequently, we cannot exclude the possibility that some of the shared immune-related signatures observed between the two cancer types may be influenced by disease progression rather than reflecting strictly cancer-type-specific biology. Advanced tumors are known to induce profound systemic immune alterations, which may contribute to overlapping circulating profiles across different malignancies. Additionally, the reported high AUC values for combined biomarker panels were derived from the same dataset may overestimate performance; external validation or resampling methods such as cross-validation are needed in future studies. Nevertheless, the primary objective of this work was to explore common circulating immune patterns in the context of an early, non-invasive multi-cancer detection strategy, rather than to perform a prognostic analysis. In this exploratory framework, the identification of shared immune dysregulation remains informative, as it reflects systemic host–tumor interactions that may be detectable across cancer types. Future prospective studies incorporating well-balanced cohorts, detailed staging information, and metastatic status are essential to disentangle cancer-specific effects from disease stage–related immune alterations.

Moreover, our results should be considered with caution since immune signatures do not operate in isolation and the tumorogenesis process can be influenced by genetic predisposition and systemic environmental or metabolic factors. In fact, a recent study has reported that an elevated blood molybdenum levels are associated with an increased risk of OC on BRCA1 mutation carriers (32).

## Conclusion

Our findings highlight the potential of a multi-protein serum biomarker panel a primordial step toward the development of a multi-cancer early detection test for improving early identification, monitoring, and personalized management of both BC and OC. While certain limitations related to the retrospective design and sample size are acknowledged, the primary focus of this study was to evaluate early diagnostic performance rather than clinicopathological stratification. These results support the relevance of immune-related circulating proteins for non-invasive detection strategies and provide a foundation for future prospective validation studies.

The robust analytical framework and inclusion of dual cancer types establish a solid foundation for future research. Further validation and integration with complementary omics data will be necessary to translate these findings into clinical practice. Ultimately, such biomarker panels may pave the way for more precise, timely, and effective therapies, addressing significant unmet needs in gynecologic oncology.

## Data Availability

The data presented in the study are deposited in the Zenodo repository, accession number 10.5281/zenodo.18980479.

## Glossary

BC: Breast cancer
OC: Ovarian cancer
HC: Healthy controls
ROC: Receiver Operating Characteristic
STRING: Search Tool for the Retrieval of Interacting Genes/Proteins
TME: tumor microenvironment
MCED: Multi-Cancer Early Detection
SAI: Salah Azaeiz Institute
PCA: Principal Component Analysis, PLS-DA, Partial Least Squares Discriminant Analysis
AUC: Area Under the Curve, G-CSF, Granulocyte Colony-Stimulating Factor, GM-CSF, Granulocyte-Macrophage Colony-Stimulating Factor
Interleukin: LIF, Leukemia Inhibitory Factor, M-CSF, Macrophage Colony-Stimulating Factor
MIF: Macrophage Migration Inhibitory Factor
TNF: Tumor Necrosis Factor
TSLP: Thymic Stromal Lymphopoietin
BLC: B lymphocyte chemoattractant (CXCL13), ENA-78, Epithelial-derived Neutrophil-Activating Peptide 78 (CXCL5), Eotaxin-1, (CCL11), Eotaxin-2, (CCL24), Eotaxin-3, (CCL26)
Fractalkine: (CX3CL1), GROα, Growth-Regulated Oncogene alpha (CXCL1), IP-10, Interferon gamma-induced protein 10 (CXCL10), I-TAC, Interferon-inducible T-cell alpha chemoattractant (CXCL11)
MCP: Monocyte Chemoattractant Protein
MDC: Macrophage-Derived Chemokine(CCL22)
MIG: Monokine Induced by Gamma interferon (CXCL9)
MIP: Macrophage Inflammatory Protein, SDF-1α, Stromal cell-derived factor 1 alpha (CXCL12), FGF-2, Fibroblast Growth Factor 2
HGF: Hepatocyte Growth Factor, MMP-1, Matrix Metalloproteinase-1, NGF-β, Nerve Growth Factor beta
SCF: Stem Cell Factor, VEGF-A, Vascular Endothelial Growth Factor A
BTLA: B and T Lymphocyte Attenuator
CD: Cluster of Differentiation, CTLA-4, Cytotoxic T-Lymphocyte Antigen 4
GITR: Glucocorticoid-Induced TNFR-Related Protein (TNFRSF18)
GITRL: GITR Ligand (TNFSF18)
HVEM: Herpesvirus Entry Mediator (TNFRSF14)
ICOS: Inducible T-cell CO-Stimulator, LAG-3, Lymphocyte Activation Gene-3, PD-1, Programmed Cell Death Protein 1, PD-L1, Programmed Death-Ligand 1, TIM-3, T-cell Immunoglobulin and Mucin-domain containing-3
TLR2: Toll-Like Receptor 2.

## References

[1] Globocan 2022 (CIRC/IARC) . Cancer incidence and mortality worldwide — Données globales sur le cancer de l’ovaire. Available online at: https://gco.iarc.fr/ (Accessed August 11, 2025).

[2] Çelik A Acar M Moroski Erkul C Gunduz E Gunduz M . Relationship of breast cancer with ovarian cancer. InTech. (2015). doi: 10.5772/59682

[3] Pawar S Liew TO Stanam A Lahiri C . Common cancer biomarkers of breast and ovarian types identified through artificial intelligence. Chem Biol Drug Des. (2020) 96:995–1004. doi: 10.1111/cbdd.13672, PMID: 32410355

[4] Norouzi-Barough L Asgari Khosro Shahi A Mohebzadeh F Masoumi L Haddadi MR Shirian S . Early diagnosis of breast and ovarian cancers by body fluids circulating tumor-derived exosomes. Cancer Cell Int. (2020) 20:187. doi: 10.1186/s12935-020-01276-x, PMID: 32489323 PMC7247259

[5] Alganmi N Bashanfar A Alotaibi R Banjar H Karim S Mirza Z . Uncovering hidden genetic risk factors for breast and ovarian cancers in BRCA-negative women: a machine learning approach in the Saudi population. PeerJ Comput Sci. (2024) 10:e1942. doi: 10.7717/peerj-cs.1942, PMID: 38660159 PMC11042021

[6] Erasha AM EL-Gendy H Aly AS Fernández-Ortiz M Sayed RKA . The role of the tumor microenvironment (TME) in advancing cancer therapies: immune system interactions, tumor-infiltrating lymphocytes (TILs), and the role of exosomes and inflammasomes. Int J Mol Sci. (2025) 26:2716. doi: 10.3390/ijms26062716, PMID: 40141358 PMC11942452

[7] Yu H Li J Peng S Liu Q Chen D He Z . Tumor microenvironment: Nurturing cancer cells for immunoevasion and druggable vulnerabilities for cancer immunotherapy. Cancer Lett. (2024) 611:217385. doi: 10.1016/j.canlet.2024.217385, PMID: 39645024

[8] Rapoport BL Steel HC Hlatshwayo N Theron AJ Meyer PWA Nayler S . Systemic immune dysregulation in early breast cancer is associated with decreased plasma levels of both soluble co-inhibitory and co-stimulatory immune checkpoint molecules. Front Immunol. (2022) 13:823842. doi: 10.3389/fimmu.2022.823842, PMID: 35677046 PMC9168983

[9] Rapoport BL Steel HC Benn CA Nayler S Smit T Heyman L . Dysregulation of systemic soluble immune checkpoints in early breast cancer is attenuated following administration of neoadjuvant chemotherapy and is associated with recovery of CD27, CD28, CD40, CD80, ICOS and GITR and substantially increased levels of PD-L1, LAG-3 and TIM-3. Front Oncol. (2023) 13:1097309. doi: 10.3389/fonc.2023.1097309, PMID: 37064132 PMC10098332

[10] Preston CC Goode EL Hartmann LC Kalli KR Knutson KL . Immunity and immune suppression in human ovarian cancer. Immunotherapy. (2011) 3:539–56. doi: 10.2217/imt.11.20, PMID: 21463194 PMC3147144

[11] Habel A Xu W Hadj Ahmed M Stayoussef M Bouaziz H Ayadi M . Identification of two theranostic biomarker panels for epithelial ovarian cancer. Cytokine. (2023) 161:156051. doi: 10.1016/j.cyto.2022.156051, PMID: 36401984

[12] Colombo N Gadducci A Landoni F Lorusso D Sabbatini R Artioli G . Consensus statements and treatment algorithm to guide clinicians in the selection of maintenance therapy for patients with newly diagnosed, advanced ovarian carcinoma: Results of a Delphi study. Gynecol Oncol. (2023) 175:182–9. doi: 10.1016/j.ygyno.2023.05.065, PMID: 37355448

[13] Imai M Nakamura Y Yoshino T . Transforming cancer screening: the potential of multi-cancer early detection (MCED) technologies. Int J Clin Oncol. (2025) 30:180–93. doi: 10.1007/s10147-025-02694-5, PMID: 39799530 PMC11785667

[14] García-Cañizares VM González-Vidal A Burgos-Molina AM Mercado-Sáenz S Sendra-Portero F Ruiz-Gómez MJ . Common molecular mechanisms and biomarkers in breast, colon and ovarian cancer. Appl Sci. (2025) 15:7018. doi: 10.3390/app15137018, PMID: 41725453

[15] Lučić I Kurtović M Mlinarić M Piteša N Čipak Gašparović A Sabol M . Deciphering common traits of breast and ovarian cancer stem cells and possible therapeutic approaches. Int J Mol Sci. (2023) 24:10683. doi: 10.3390/ijms241310683, PMID: 37445860 PMC10342190

[16] Balkwill F . Cancer and the chemokine network. Nat Rev Cancer. (2004) 4:540–50. doi: 10.1038/nrc1388, PMID: 15229479

[17] Moura T Laranjeira P Caramelo O Gil AM Paiva A . Breast cancer and tumor microenvironment: the crucial role of immune cells. Curr Oncol. (2025) 32:143. doi: 10.3390/curroncol32030143, PMID: 40136347 PMC11941043

[18] Sharma O Sultan AA Ding H Triggle CR . A review of the progress and challenges of developing a vaccine for COVID-19. Front Immunol. (2020) 11:585354. doi: 10.3389/fimmu.2020.585354, PMID: 33163000 PMC7591699

[19] Sarvaiya PJ Guo D Ulasov I Gabikian P Lesniak MS . Chemokines in tumor progression and metastasis. Oncotarget. (2013) 4:2171–85. doi: 10.18632/oncotarget.1426, PMID: 24259307 PMC3926818

[20] Ghalehbandi S Yuzugulen J Pranjol MZI Pourgholami MH . The role of VEGF in cancer-induced angiogenesis and research progress of drugs targeting VEGF. Eur J Pharmacol. (2023) 949:175586. doi: 10.1016/j.ejphar.2023.175586, PMID: 36906141

[21] Grillo E Romani C Ettorre VM Santin AD Mitola S . The VEGF/VEGFR2 system in ovarian cancer: From functional to pharmacological significance. Biochim Biophys Acta Rev Cancer. (2025) 1880:189374. doi: 10.1016/j.bbcan.2025.189374, PMID: 40516635

[22] Moskov M Hedlund Lindberg J Lycke M Ivansson E Gyllensten U Sundfeldt K . Deep plasma proteomics identifies and validates an eight-protein biomarker panel that separate benign from Malignant tumors in ovarian cancer. Commun Med. (2025) 5:230. doi: 10.1038/s43856-025-00945-0, PMID: 40506476 PMC12162877

[23] Muinao T Deka Boruah HP Pal M . Multi-biomarker panel signature as the key to diagnosis of ovarian cancer. Heliyon. (2019) 5:e02826. doi: 10.1016/j.heliyon.2019.e02826, PMID: 31867451 PMC6906658

[24] Wang Y Zhu N Liu J Chen F Song Y Ma Y . Role of tumor microenvironment in ovarian cancer metastasis and clinical advancements. J Transl Med. (2025) 23:539. doi: 10.1186/s12967-025-06508-0, PMID: 40369674 PMC12079989

[25] Yoneda A Lendorf ME Couchman JR Multhaupt HA . Breast and ovarian cancers: a survey and possible roles for the cell surface heparan sulfate proteoglycans. J Histochem Cytochem. (2012) 60:9–21. doi: 10.1369/0022155411428469, PMID: 22205677 PMC3283135

[26] Abdul-Rahman T Ghosh S Badar SM Nazir A Bamigbade GB Aji N . The paradoxical role of cytokines and chemokines at the tumor microenvironment: a comprehensive review. Eur J Med Res. (2024) 29:124. doi: 10.1186/s40001-024-01711-z, PMID: 38360737 PMC10868116

[27] Budhwani M Turrell G Yu M Frazer IH Mehdi AM Chandra J . Immune-inhibitory gene expression is positively correlated with overall immune activity and predicts increased survival probability of cervical and head and neck cancer patients. Front Mol Biosci. (2021) 8:622643. doi: 10.3389/fmolb.2021.622643, PMID: 33834038 PMC8021786

[28] Palacios-Arreola MI Nava-Castro KE Castro JI García-Zepeda E Carrero JC Morales-Montor J . The role of chemokines in breast cancer pathology and its possible use as therapeutic targets. J Immunol Res. (2014) 2014:849720. doi: 10.1155/2014/849720, PMID: 25165728 PMC4139084

[29] Pazoki A Dadfar S Shadab A Haghmorad D Oksenych V . Soluble CD40 ligand as a promising biomarker in cancer diagnosis. Cells. (2024) 13:1267. doi: 10.3390/cells13151267, PMID: 39120299 PMC11311304

[30] Yang Y Li J Lei W Wang H Ni Y Liu Y . CXCL12-CXCR4/CXCR7 axis in cancer: from mechanisms to clinical applications. Int J Biol Sci. (2023) 19:3341–59. doi: 10.7150/ijbs.82317, PMID: 37497001 PMC10367567

[31] Zielińska KA Katanaev VL . The signaling duo CXCL12 and CXCR4: chemokine fuel for breast cancer tumorigenesis. Cancers. (2020) 12:3071. doi: 10.3390/cancers12103071, PMID: 33096815 PMC7590182

[32] Matuszczak M Kiljańczyk A Marciniak W Derkacz R Stempa K Baszuk P . Blood molybdenum level as a marker of cancer risk on BRCA1 carriers. Hereditary Cancer Clin Pract. (2024) 22:19. doi: 10.1186/s13053-024-00291-7, PMID: 39300540 PMC11411732

