# Supplementary material for: Breast and ovarian cancers: toward a multi-cancer early detection test
Source: Front Immunol. 2026 Mar 19;17:1741582. doi: 10.3389/fimmu.2026.1741582 (PMC13044938; doi:10.3389/fimmu.2026.1741582)
Supplement: Supplementary file 1 [file Table1.docx]

Supplementary Material

# Supplementary table 1. Comparative analyses among breast cancer, ovarian cancer, and healthy control groups

| **Protein** | **Mean_Control** | **SD_Control** | **Mean_BC** | **SD_BC** | **Mean_OC** | **SD_OC** | **p1** | **p2** | **p3** |
| --- | --- | --- | --- | --- | --- | --- | --- | --- | --- |
| **Fractalkine** | 24.22 | 15.50 | 47.23 | 24.81 | 36.16 | 15.08 | <10-3 | <10-3 | 0.01 |
| **VEGF-A** | 717.06 | 663.31 | 1633.47 | 1939.64 | 1054.32 | 742.92 | <10-3 | 0.01 | 0.04 |
| **BLC** | 501.43 | 1450.47 | 103.30 | 87.65 | 98.27 | 70.32 | <10-3 | <10-3 | 0.76 |
| **Eotaxin** | 89.37 | 87.77 | 34.20 | 35.87 | 30.46 | 38.16 | <10-3 | <10-3 | 0.22 |
| **IP-10** | 90.58 | 78.30 | 125.72 | 439.75 | 52.03 | 63.90 | <10-3 | <10-3 | 0.33 |
| **SDF-1A** | 1789.26 | 1761.89 | 2134.28 | 5105.77 | 1238.97 | 604.27 | 0.02 | <10-3 | 0.40 |
| **CD30** | 559.98 | 468.44 | 757.81 | 555.04 | 932.98 | 738.63 | 0.02 | <10-3 | 0.21 |
| **IL-2R** | 3942.66 | 3366.81 | 5415.38 | 5698.77 | 5163.13 | 4000.25 | 0.04 | 0.04 | 0.72 |
| **TNFRII** | 252.74 | 108.77 | 328.95 | 167.68 | 290.20 | 69.20 | <10-3 | 0.03 | 0.29 |
| **Tweak** | 2402.05 | 1904.51 | 2665.72 | 9844.51 | 1452.48 | 2187.51 | <10-3 | <10-3 | 0.27 |
| **MMP-1** | 412.09 | 293.60 | 251.03 | 181.72 | 322.28 | 355.75 | <10-3 | 0.01 | 0.85 |
| **BTLA** | 142.75 | 197.67 | 100.53 | 163.58 | 84.33 | 119.70 | 0.03 | 0.02 | 0.54 |
| **CD40** | 758.57 | 524.97 | 1057.45 | 683.00 | 1031.00 | 1020.11 | 0.03 | <10-3 | 0.98 |
| **CD80/B7-1** | 20.21 | 19.14 | 15.31 | 22.34 | 15.62 | 26.81 | 0.02 | 0.01 | 0.71 |
| **GITRL** | 65.42 | 50.76 | 45.23 | 53.92 | 53.81 | 100.70 | 0.03 | 0.01 | 0.97 |
| **LAG-3** | 111703.96 | 115972.67 | 7272.46 | 5914.15 | 29854.71 | 57907.46 | <10-3 | <10-3 | 0.62 |
| **APRIL** | 2253.31 | 1230.06 | 2038.14 | 1663.90 | 1658.02 | 846.33 | 0.04 | <10-3 | 0.07 |
| **CD40-Ligand** | 246.60 | 258.65 | 349.51 | 1340.03 | 181.55 | 206.73 | 0.04 | <10-3 | 0.42 |
| **IFN-gamma** | 13.14 | 7.04 | 12.42 | 7.30 | 11.11 | 9.31 | 0.32 | 0.01 | 0.02 |
| **IL-12p70** | 2.87 | 1.42 | 2.76 | 1.71 | 2.30 | 1.23 | 0.47 | 0.01 | 0.01 |
| **IL-16** | 178.34 | 163.62 | 296.74 | 395.53 | 150.04 | 131.06 | 0.08 | 0.17 | <10-3 |
| **IL-18** | 38.25 | 20.69 | 43.83 | 31.45 | 31.54 | 18.01 | 0.86 | 0.04 | 0.03 |
| **IL-1alpha** | 9.54 | 13.73 | 28.64 | 109.13 | 8.64 | 15.84 | 0.01 | 0.24 | <10-3 |
| **IL-1beta** | 9.12 | 9.04 | 43.98 | 93.05 | 25.29 | 103.82 | <10-3 | 0.26 | <10-3 |
| **IL-2** | 39.37 | 49.29 | 44.26 | 32.42 | 33.70 | 29.24 | 0.06 | 0.88 | 0.02 |
| **IL-21** | 59.81 | 69.29 | 207.29 | 972.47 | 51.03 | 48.08 | 0.01 | 1.00 | <10-3 |
| **IL-22** | 99.59 | 208.71 | 107.22 | 233.47 | 58.45 | 63.56 | 0.22 | 0.37 | 0.01 |
| **IL-8** | 19.20 | 16.73 | 371.20 | 872.11 | 132.56 | 473.26 | 0.02 | 0.90 | 0.02 |
| **LIF** | 2.29 | 2.74 | 4.80 | 7.08 | 2.16 | 1.92 | 0.04 | 0.76 | 0.03 |
| **M-CSF** | 35.53 | 38.79 | 48.67 | 51.58 | 29.71 | 42.44 | 0.06 | 0.13 | <10-3 |
| **MIF** | 120.65 | 75.24 | 186.86 | 120.11 | 118.63 | 77.77 | <10-3 | 0.72 | <10-3 |
| **TNF-alpha** | 10.47 | 5.98 | 10.56 | 7.41 | 11.62 | 22.22 | 0.71 | <10-3 | <10-3 |
| **TSLP** | 16.71 | 15.78 | 15.69 | 19.17 | 11.05 | 10.05 | 0.48 | 0.01 | 0.01 |
| **Eotaxin-3** | 20.39 | 8.57 | 24.04 | 8.59 | 17.97 | 9.20 | 0.02 | 0.09 | <10-3 |
| **I-TAC** | 104.19 | 179.39 | 57.54 | 51.60 | 49.77 | 63.57 | 0.70 | 0.05 | 0.06 |
| **MIP-1beta** | 84.00 | 48.26 | 221.48 | 463.08 | 101.82 | 172.47 | 0.02 | 0.08 | <10-3 |
| **HGF** | 440.79 | 354.00 | 537.05 | 405.36 | 373.36 | 225.87 | 0.08 | 0.47 | 0.01 |
| **SCF** | 20.08 | 10.57 | 22.07 | 14.22 | 18.15 | 12.54 | 0.61 | 0.19 | 0.04 |
| **TIM-3** | 3682.71 | 2370.53 | 3927.21 | 2292.44 | 4752.79 | 2902.88 | 0.57 | 0.01 | 0.04 |
| **TLR-2** | 690.12 | 676.23 | 942.97 | 3300.39 | 601.17 | 974.13 | 0.33 | <10-3 | 0.01 |
| **TRAIL** | 60.07 | 92.16 | 232.80 | 1145.85 | 44.20 | 36.73 | 0.02 | 0.49 | <10-3 |
| **Eotaxin-2** | 770.05 | 553.11 | 523.48 | 190.36 | 487.91 | 241.56 | 0.14 | 0.05 | 0.26 |
| **GITR** | 74.77 | 147.48 | 63.65 | 29.20 | 59.99 | 46.79 | 0.02 | 0.43 | 0.19 |
| **G-CSF/CSF-3** | 16.45 | 14.62 | 28.64 | 46.19 | 62.29 | 140.11 | 0.98 | 0.23 | 0.37 |
| **GM-CSF** | 24.32 | 14.82 | 18.48 | 12.75 | 33.45 | 19.09 | 0.33 | 0.54 | 0.09 |
| **IFN-alpha** | 1.29 | 0.84 | 29.90 | 42.85 | 5.84 | 4.13 | 0.11 | 0.13 | 0.53 |
| **IL-10** | 8.72 | 20.88 | 2.73 | 4.47 | 2.40 | 4.65 | 0.19 | 0.13 | 0.32 |
| **IL-13** | 4.28 | 4.26 | 5.55 | 11.99 | 4.02 | 3.89 | 0.60 | 0.61 | 0.88 |
| **IL-15** | 10.45 | 19.57 | 10.86 | 18.09 | 10.45 | 14.75 | 0.87 | 0.85 | 0.98 |
| **IL-17A** | 40.05 | 49.76 | 54.12 | 182.75 | 36.04 | 61.04 | 0.50 | 0.75 | 0.81 |
| **IL-20** | 38.73 | 87.88 | 64.13 | 279.15 | 34.45 | 80.77 | 0.25 | 0.48 | 0.71 |
| **IL-23** | 40.77 | 115.96 | 13.16 | 12.43 | 17.55 | 28.41 | 0.44 | 0.67 | 0.48 |
| **IL-27** | 20.55 | 20.92 | 68.46 | 191.45 | 33.62 | 48.64 | 0.62 | 0.87 | 0.46 |
| **IL-3** | 78.37 | 159.32 | 78.79 | 158.96 | 52.04 | 72.50 | 0.40 | 0.73 | 0.64 |
| **IL-31** | 42.46 | 127.21 | 23.24 | 38.71 | 40.21 | 76.64 | 0.90 | 0.43 | 0.62 |
| **IL-4** | 26.91 | 7.18 | 31.35 | 34.55 | 102.94 | 138.59 | 0.63 | 0.70 | 0.50 |
| **IL-5** | 13.93 | 15.72 | 8.82 | 9.88 | 4.80 | 4.77 | 0.82 | 0.48 | 0.12 |
| **IL-6** | 36.64 | 47.26 | 196.40 | 670.17 | 119.22 | 276.09 | 0.51 | 0.94 | 0.60 |
| **IL-7** | 3.59 | 2.78 | 4.59 | 6.41 | 3.74 | 1.91 | 0.32 | 0.21 | 0.97 |
| **IL-9** | 11.71 | 18.29 | 26.05 | 29.48 | 41.53 | 32.27 | 0.19 | 0.09 | 0.31 |
| **TNF-beta** | 45.16 | 91.47 | 10.14 | 12.77 | 33.61 | 66.32 | 0.61 | 0.57 | 0.93 |
| **ENA-78 (LIX)** | 1353.49 | 1376.99 | 113<10-3 | 1079.94 | 890.13 | 620.16 | 0.37 | 0.14 | 0.58 |
| **Gro-alpha** | 13.25 | 16.80 | 18.16 | 25.12 | 16.50 | 40.64 | 0.09 | 0.84 | 0.07 |
| **MCP-1** | 170.49 | 104.76 | 281.02 | 347.75 | 312.23 | 494.25 | 0.45 | 0.12 | 0.54 |
| **MCP-2** | 29.93 | 20.04 | 23.36 | 14.60 | 21.97 | 10.18 | 0.08 | 0.07 | 0.97 |
| **MCP-3** | 13.28 | 4.98 | 17.40 | 13.11 | 69.78 | 200.97 | 0.43 | 0.90 | 0.58 |
| **MDC/CCL22** | 452.42 | 238.08 | 452.43 | 199.50 | 432.69 | 214.04 | 0.89 | 0.88 | 0.84 |
| **MIG** | 155.37 | 156.35 | 348.84 | 1234.02 | 148.68 | 158.73 | 0.20 | 1.00 | 0.16 |
| **MIP-1alpha** | 23.83 | 29.67 | 69.47 | 176.64 | 35.59 | 72.09 | 0.37 | 0.75 | 0.24 |
| **MIP-3alpha** | 34.15 | 24.60 | 74.60 | 173.75 | 44.23 | 80.03 | 0.98 | 0.54 | 0.49 |
| **BAFF** | 7.57 | 7.36 | 10.84 | 20.77 | 9.60 | 17.30 | 0.68 | 0.26 | 0.05 |
| **FGF-2** | 13.90 | 24.48 | 13.36 | 12.36 | 12.56 | 16.85 | 0.16 | 0.45 | 0.19 |
| **bNGF** | 18.50 | 17.81 | 20.03 | 23.94 | 29.16 | 47.95 | 0.85 | 0.89 | 0.88 |
| **CD27** | 4084.70 | 5630.40 | 3910.61 | 4401.88 | 4516.04 | 6194.52 | 0.68 | 0.14 | 0.36 |
| **CD28** | 1071.26 | 1860.53 | 1125.87 | 1936.23 | 1952.97 | 7465.35 | 0.49 | 0.63 | 0.96 |
| **CD86/B7-2** | 328.80 | 208.09 | 341.57 | 183.23 | 342.13 | 287.82 | 0.64 | 0.55 | 0.30 |
| **CTLA-4** | 14.73 | 18.20 | 10.85 | 9.56 | 13.87 | 21.57 | 0.23 | 0.69 | 0.30 |
| **HVEM** | 3718.35 | 1779.80 | 4647.22 | 2988.63 | 4176.91 | 3054.06 | 0.14 | 0.46 | 0.36 |
| **ICOS** | 128.95 | 155.79 | 106.30 | 138.38 | 129.19 | 176.10 | 0.43 | 0.49 | 0.99 |
| **PD-1** | 380.45 | 375.32 | 540.57 | 1901.45 | 386.30 | 386.62 | 0.13 | 0.99 | 0.07 |
| **PD-L1** | 23.44 | 16.52 | 19.17 | 11.83 | 24.98 | 32.11 | 0.15 | 0.69 | 0.19 |

Comparisons were performed using the Mann–Whitney test. p =pvalue, p1: BC vs. HC; p2: OC vs. HC; p3: BC vs. OC, SD: Stand of deviations

Cells highlighted in grey indicate significant differences among all three groups (BC-HC, OC-HC, and BC-OC).

Yellow cells indicate significant differences between one or two groups, while green cells indicate no significant difference.
